# Progress of our journal at glance

**DOI:** 10.4103/0974-7796.62911

**Published:** 2010

Dear All,

It's my pleasure to write to you the first issue of our second volume. Last year had been very fruitful for us as we published our first volume with two issues in it. We are started to be known locally and internationally, and we have a large bank of urologist worldwide for electronic communications with more than 200 reviewers, from different countries, actively participating in the peer review process.

Till date, we've received more than 160 manuscripts and our acceptance rate has been around 39%. We are planning to have three issues this year and the next year, and by 2012, we shall reach our goal of four issues per year.

We are very optimistic on the growth of our journal, from the enthusiasm and the support towards our new journal, and we thus hope to have a strong presence in the near future.

We are also planning to start communication by next year with all the regional universities to accredit our journal by their scientific councils.

Once more, I extend my invitation to all urologists to join us in the success of our journal.

Yours truly,


Dr. Khalid Fouda Neel
Associate Professor of Urology,
Editor-in-Chief,
Urology Annals.
E-mail: kfouda@ksu.edu.sa


